# Phenylketonuria Alters the Prefrontal Cortex Genome-Wide Expression Profile Regardless of the Mouse Genetic Background

**DOI:** 10.3390/cells15030227

**Published:** 2026-01-24

**Authors:** Elena Fiori, Serafina Manila Guzzo, Luisa Lo Iacono, Cristina Orsini, Simona Cabib, Diego Andolina, Luigia Rossi, Francesca Nardecchia, Vincenzo Leuzzi, Tiziana Pascucci

**Affiliations:** 1 Technopole Foundation, 00185 Rome, Italy; elena.fiori@uniroma1.it; 2 Department of Psychology, Centro “Daniel Bovet”, Sapienza University, 00185 Rome, Italy; serafinamanila.guzzo@uniroma1.it (S.M.G.); cristina.orsini@uniroma1.it (C.O.); diego.andolina@uniroma1.it (D.A.); 3 Department of Translational Research and of New Surgical and Medical Technologies, University of Pisa, 56126 Pisa, Italy; luisa.loiacono@unipi.it; 4 Fondazione Santa Lucia Istituto di Ricovero e Cura a Carattere Scientifico, 00143 Rome, Italy; s.cabib@hsantalucia.it; 5 Department of Biomolecular Science, University of Urbino, 61029 Urbino, Italy; luigia.rossi@uniurb.it; 6 Unit of Child Neurology and Psychiatry, Department of Human Neuroscience, Sapienza University of Rome, 00185 Rome, Italy; francesca.nardecchia@uniroma1.it (F.N.); vincenzo.leuzzi@fondazione.uniroma1.it (V.L.)

**Keywords:** phenylketonuria, prefrontal cortex, transcriptome, HPA resilience and vulnerability, attention and memory

## Abstract

Mouse models of genetic diseases are important research tools. However, the genetic background of the mouse strain can significantly influence how a genetic mutation is expressed. Studies on preclinical models of phenylketonuria (PKU), an inherited metabolic disorder, have used two strains, BTBR and C57Bl/6, created via a chemically induced point mutation in the gene encoding the enzyme phenylalanine hydroxylase (BTBR^enu2^ and C57^enu2^, respectively). Despite having the same levels of hyperphenylalaninemia (HPA), published results indicate differences in neural and behavioral phenotypes between the two backgrounds. To explore this difference further, the current study examines the genome-wide transcriptome of the prefrontal cortex (pFC), the brain region which is the most vulnerable to the negative effects of HPA. Regardless of the strain, the enu2 mutation upregulated the expression of several aminoacyl-tRNA synthetases and eukaryotic translation initiation factors, suggesting an essential modification in the protein translation process and supporting the downregulation of gene programs related to myelination. Accordingly, we deepened the exploration of cognitive dysfunctions in C57^enu2−^ mice, showing a previously unreported working memory impairment under increasing information load. These findings identify convergent pFC molecular and cognitive alterations induced by HPA across distinct genetic backgrounds, providing clinically relevant insights into mechanisms that may contribute to executive dysfunctions in PKU.

## 1. Introduction

Phenylketonuria (PKU; OMIM #261600) is an inherited metabolic disorder caused by a defect in the phenylalanine hydroxylase (PAH) enzyme (EC 1.14.16.1), which converts phenylalanine (Phe) into tyrosine (Tyr). The resulting accumulation of Phe (hyperphenylalaninemia, HPA) in biological fluids and brain tissue leads to severe neurodevelopmental and neurological derangements. Neonatal screening programs for HPA, enabling presymptomatic diagnosis and early treatment of children with PKU, have been successful over the past 60 years in preventing the severe consequences of the disease in thousands of children. However, under current therapies, early-treated PKU (ETPKU) patients as a group tend to have an intellectual quotient (IQ) gap compared to their non-PKU siblings or unaffected controls, show mild impairment in neuropsychological functions—particularly in executive functions—and face an increased risk of psychiatric illness [1,2,3]. Finally, there is a wide variability in clinical outcomes among young adults, regardless of their level of metabolic control, and the long-term effects of Phe exposure on the brain functions of older patients still require further study.

These findings sparked interest in exploring the direct effects of HPA on brain development and function using animal models of metabolic disease. Most studies focused on BTBR^enu2^ mice, generated through a chemically induced genetic mutation [4] in the BTBR inbred background. BTBR^enu2^ exhibits severe HPA (blood Phe levels 10–20 times higher than those in wild-type littermates), mimicking the classic biochemical phenotype of PKU in humans. Significant somatic and neurological changes were observed in both developing and adult BTBR^enu2^ mice [5,6,7,8,9,10,11]. Interestingly, the phenotypic expression of the PAH^enu2^ mutation in the standard C57BL/6 mouse background (C57^enu2^), which also experiences severe HPA, does not produce the same severe behavioral phenotype observed in BTBR^enu2^ mice [12], raising questions about the translational relevance of these mouse models. Despite the large clinical variability among untreated subjects with PKU (and also observed in early-treated adults), the occurrence of asymptomatic individuals is a rare event. However, the occurrence of such a different outcome in two biochemically overlapping preclinical models may help reveal previously unknown biological factors involved in the disease outcome.

In this context, as the initial step, we conducted a genome-wide transcriptome analysis of prefrontal cortex (pFC) samples collected from adult PKU and wild-type mice of two backgrounds. pFC functions seem to be especially sensitive to early and late exposure to HPA in PKU patients [1,2,3]. Based on the results of this first study, we examined pFC functions more deeply in C57^enu2^ untreated adult mice using a more cognitively demanding task [13,14,15]. The inability of BTBR^enu2^ mice to recognize novelty in the presence of only two objects is well known [5,6,7,8], requiring no further confirmation. Instead, we stressed the cognitive abilities of C57^enu2^ mice by increasing the number of objects present in the object recognition test up to six objects simultaneously.

## 2. Materials and Methods

### 2.1. Animals

Two different strains, the Black and Tan Brachyury (BTBR) and the C57Bl/6JRj (C57), were used as wild-type (WT) controls (hereafter referred to as C57 and BTBR) along with homozygous ^−/−^ knock-out mutant mice, where ethyl nitrosourea (ENU) induced the same mutation in the gene encoding for PAH in both strains (C57^enu2^ and BTBR^enu2^ mice). Animals were raised in the animal facility at the Psychology Department of Sapienza University. Experimental (ENU) and control (WT) groups were obtained through heterozygous mating, and sex-matched littermates were housed 3–4 per standard cage after weaning, with a 12 h light/dark cycle in a temperature-controlled environment (21 °C). Standard food (Mucedola 4RF21 Certificate) and water were provided ad libitum. All animals were provided with the same standard environmental enrichment. Genetic characterization was performed in both strains as previously described [4]. Both male and female mice were used in all experiments. The stage of the cycle was not measured in female groups. All experiments were conducted in accordance with the European legislation (2010/63/EU) and with the Italian national legislation (DL26/2014), which regulates the use of animals for research according to the guidelines of the National Institute of Health on the use and the care of laboratory animals (Authorization n° 485/2021-PR).

### 2.2. Brain Punches and RNA Extraction

Two-month-old naive C57^enu2^, BTBR^enu2^, and control mice were sacrificed between 12:00 and 13:00 during the light phase of the light–dark cycle (ZT5–ZT6). Brains were rapidly collected and stored at −80 °C. Micro-punches of the prefrontal cortex (pFC) were obtained from two coronal brain sections (300 μm thickness) using a stainless steel tube with an internal diameter of 1 mm. The anatomical coordinates were determined according to the Franklin and Paxinos mouse brain atlas (+1.50 to +2.10 mm from bregma). Tissue samples were stored at −80 °C until the day of the assay. Total RNA was extracted using the Total RNA Purification Kit (Norgen Biotek, Thorold, Canada) following the manufacturer’s instructions. RNA concentration was determined by measuring absorbance at 260 nm using a NanoDrop UV–Vis spectrophotometer.

### 2.3. Library Preparation, mRNA Sequencing, and Analysis

Transcriptomic analysis of mRNA samples obtained from pFC punches was performed by IGA Technology Services. Samples were collected from the following experimental groups: BTBR (4 males and 3 females), BTBR^enu2^ (3 males and 3 females), C57 (5 males and 3 females), and C57^enu2^ (4 males and 4 females). Individual RNA samples (~500 ng each) were quantified and assessed for quality using the Agilent 2100 Bioanalyzer or TapeStation systems (Agilent Technologies, Santa Clara, CA, USA). Only samples with an RNA Integrity Number (RIN) greater than 7.0 were included in downstream analyses. Library preparation was performed using the Universal Plus mRNA-Seq kit (Tecan Genomics, Redwood City, CA, USA) according to the manufacturer’s instructions (library type: fr-secondstrand). Final libraries were quantified using a Qubit 3.0 Fluorometer (Invitrogen, Carlsbad, CA, USA) and quality-checked with the Agilent Bioanalyzer DNA assay. Libraries were pooled and sequenced in paired-end mode (2 × 150 bp) on a NovaSeq 6000 system (Illumina, San Diego, CA, USA) at an average depth of 30 million reads per sample. Base calling and demultiplexing were carried out using Illumina BCL Convert v3.9.31. Adapter sequences and low-quality bases were removed using Cutadapt v1.11. Trimmed reads were aligned to the GRCm39.112 reference genome using STAR with default parameters. Quality control of aligned reads was performed using FastQC. Gene expression levels were quantified as FPKM values, and differential expression analysis was conducted using the Bioconductor DESeq2 package (version 1.34.0, Bioconda distribution). Genes with an FDR-corrected *p*-value < 0.05 were considered differentially expressed (DEGs). For pathway enrichment analyses, the DEG list was extended using a nominal *p*-value threshold of <0.01. Gene ontology and pathway analyses were performed using Ingenuity Pathway Analysis (QIAGEN, Redwood City, CA, USA). All libraries were prepared in a single batch using identical protocols and reagents and pooled prior to sequencing. Sequencing was performed across two independent runs, with a balanced distribution of reads per sample. Potential batch effects related to sequencing runs were evaluated through exploratory analyses, including principal component analysis, and no run-specific clustering was observed.

### 2.4. Behavioral Assessment

Behavioral assessment was performed on a novel group of 4-month-old C57^enu2^ mice compared with the control group. All testing sessions were videotaped using a camera placed above the apparatus and analyzed by the Video-based EthoVision System (Noldus, The Netherlands). Tests took place at the same time of the day; the same experimenter was blind to the genetic status of the mice (WT or KO); and male and female mice were tested on different days to avoid exposure to odors. In the two weeks preceding testing, mice were acclimated to the testing room and the experimenter through daily handling. On the testing day, mice were individually placed in a holding cage for 15 min and then subjected to the test.

Identical Object Task (IOT): The IOT test was performed as described for the control task in the IOT/DOT test by Sannino et al., 2012 [13]. The DOT/IOT test is a modified version of the novel object recognition test (frequently used in BTBR^enu2^ mice) in which the number of objects to remember varies, allowing the measurement of different Working Memory Capacity (WMC). Firstly, mice were introduced into the center of an empty apparatus (49 × 49 cm) and left to explore the Open Field (OF) for 10 min. Distance moved, velocity, and total exploration were analyzed as indices of motor integrity, and immobility and time in the center of the arena as indices of anxiety. Then, animals were placed in the resting cage, and after 1 min, they underwent a study phase (pretest session) during which they were exposed to four identical objects (4-IOT) or six identical objects (6-IOT) for 35 s or up to 5 min. In the test phase, one of the identical objects was replaced with a new one (whose position was changed across each mouse) and the time spent exploring the new vs. the old objects was evaluated as a measure of recognition and working memory [13,14,15]. Moreover, a recognition index [(expl new/tot expl) × 100] has been calculated to evaluate novel object preference exploration [16]. The mice that explored for less than 5 s were excluded. No other exclusion parameters were applied.

According to the reported memory capacity in the C57 strain, the control group of healthy C57 mice (N = 10) was tested in the IOT with six objects (6-IOT). As the ability of C57^enu2^ mice to recognize novelty in the presence of two objects is well known [15], we tested them with progressively more demanding 4-IOT (N = 8) and 6-IOT (N = 8) objects.

### 2.5. Statistical Analysis

For behavioral assessment, a *t*-test was performed between males and females for every task. Since no significant difference was found, mice were grouped together for statistical analysis of genotype. A T test was performed for the parameters considered in the OF test to evaluate the difference between WT and enu2.

For statistical analysis of novel object preference exploration in the recognition index [(expl new/tot expl) × 100], the Kolmogorov–Smirnov test was performed to assess normal distribution in WT and mutant mice in the 4- and 6-IOT. ENU2 mice tested on 6-IOT did not pass the test (WT = 0.2047, *p* > 0.1; ENU2 6-IOT = 0.2746, *p* = 0.0314; ENU2 4-IOT = 0.1715, *p* > 0.1); we therefore performed a non-parametric analysis using the Kruskal–Wallis test.

The Friedman test for repeated measures was used for the analysis of time spent exploring the objects in the IOT test for each group. A Dunn multiple comparison test was used when appropriate. All data were analyzed using GraphPad Prism v.10.

## 3. Results

### 3.1. Transcriptomic Responses to enu2 Mutation Are Largely Conserved Across C57 and BTBR Backgrounds

**Figure 1:**
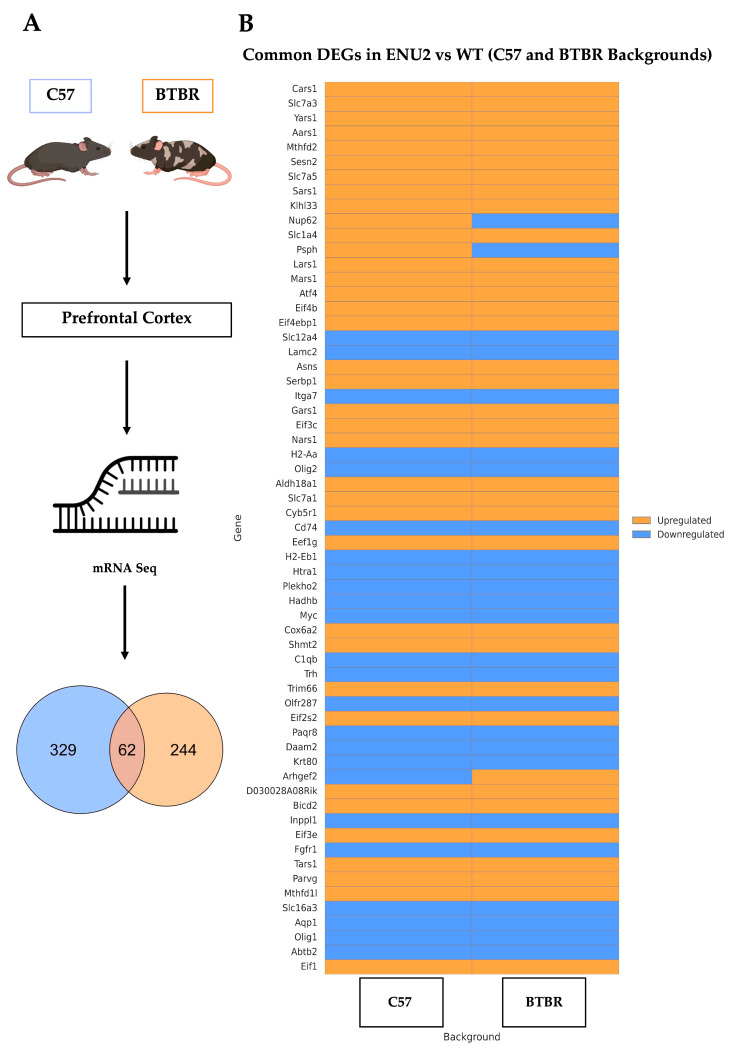
Transcriptomic analysis reveals a conserved set of differentially expressed genes (DEGs) between C57^enu2^ and BTBR^enu2^ mice. (**A**) Experimental plan and Venn diagram showing the overlap between DEGs identified in C57 (n = 391) and BTBR (n = 306) mice. DEGs were defined using an FDR-corrected *p*-value (Padj < 0.05). (**B**) Heatmap of the 62 overlapping DEGs between enu2 mutations on C57 and BTBR backgrounds. Genes are ordered by hierarchical clustering. Blue indicates downregulation and orange indicates upregulation relative to WT controls. While most genes are regulated similarly in both strains, Nup62, Psph, and Arhgef2 are regulated in opposite directions.

To investigate the impact of genetic background on transcriptional alterations associated with the enu2 mutation, we performed mRNA-Seq analysis of the pFC in adult enu2 and WT mice on both C57 and BTBR genetic backgrounds (Figure 1A). Differential expression analysis identified a total of 391 differentially expressed genes (DEGs) in the C57 background (Table S1) and 306 DEGs in the BTBR background (Table S2) (adjusted *p* < 0.05).

To identify gene transcripts commonly affected by the enu2 mutation across genetic backgrounds, we considered genes showing differential expression in each strain using a nominal *p* < 0.01 threshold and examined the overlap between the two DEG lists. This analysis revealed 62 genes that were commonly modulated in both backgrounds (Figure 1A), corresponding to approximately 16% of C57 DEGs and 20% of BTBR DEGs.

Although the proportion of overlapping DEGs represents a relatively limited fraction of the total transcriptional changes observed in each background, the shared genes were among the most significantly altered and exhibited a high degree of directional consistency. Indeed, the vast majority of common DEGs were either upregulated or downregulated in both strains, indicating conserved transcriptional responses to the enu2 mutation. Only three genes—Nup62, Psph, and Arhgef2—displayed opposite regulation patterns between the BTBR and C57 backgrounds.

The overlapping DEGs are visualized in the heatmap shown in Figure 1B and include, among others, amino acid and metabolite transporters (Slc7a3, Slc7a5, and Slc16a3), translation-related genes (Aars1, Yars1, and Eif4b), and metabolic enzymes (Mthfd1, Asns, and Psph). Importantly, while the sets of DEGs identified in each background differ in composition, gene ontology analysis of all DEGs revealed convergence on several common functional pathways, supporting the presence of conserved molecular mechanisms underlying the transcriptional effects of the enu2 mutation.

### 3.2. Activation of Translational and Metabolic Pathways in enu2 Mice Across Genetic Backgrounds

**Figure 2:**
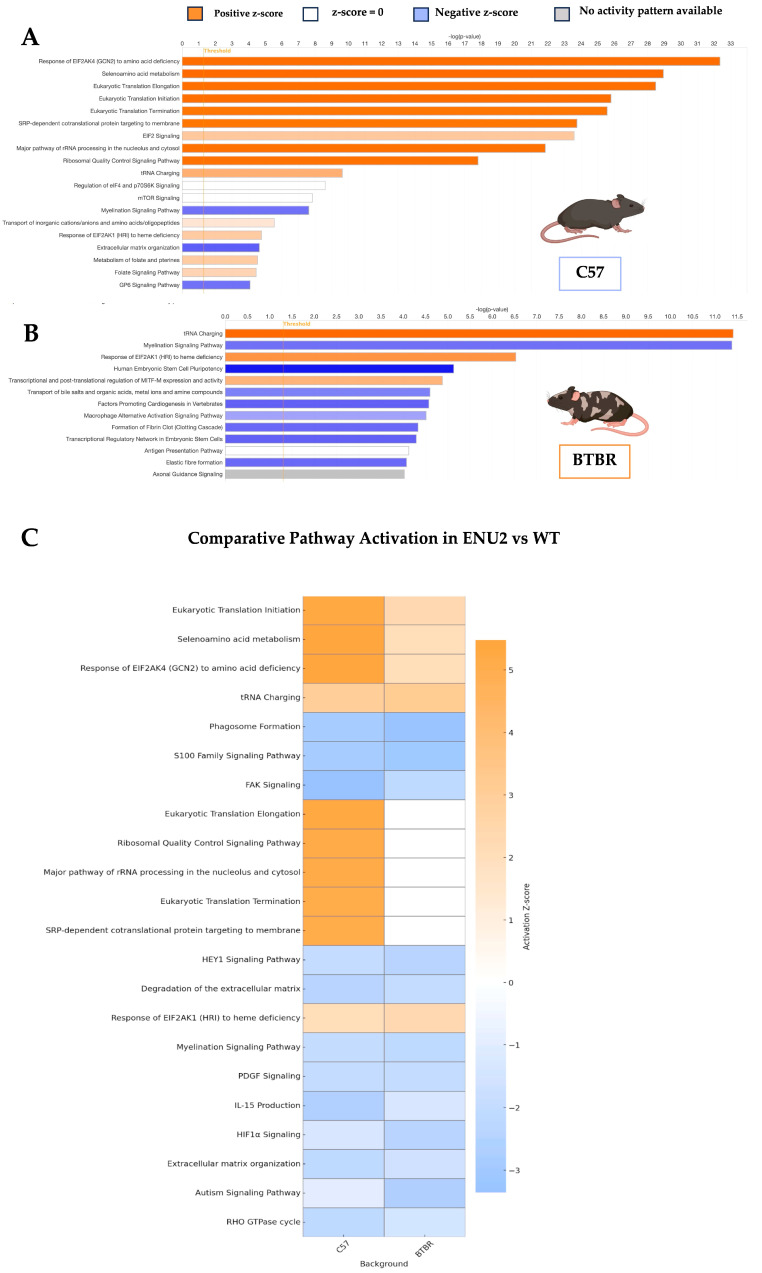
Pathway enrichment analysis in the pFC of PKU mice. (**A**) Top significantly enriched biological pathways (*p* < 0.05) in PKU mice compared to WT on the C57 background. (**B**) Top significantly enriched biological pathways (*p* < 0.05) in PKU mice compared to WT on the BTBR background. (**C**) Comparison of pathway activation between the C57 and BTBR backgrounds in PKU mice vs. WT. Bar length represents −log(p), and bar color indicates predicted activation state based on z-score: in orange are upregulated pathways (positive z-score), in blue are downregulated pathways (negative z-score), in gray there is no activity pattern available. Pathway enrichment analysis was performed using a DEG list generated with a nominal *p*-value threshold of *p* < 0.01.

To explore the biological functions influenced by the enu2 mutation, we first performed pathway enrichment analysis on the extended list of DEGs (*p* < 0.01) from individual genetic backgrounds and then analyzed the shared biological impact. In the C57 background, pathway analysis suggested a robust activation of translation-related processes (Figure 2A). Indeed, among the top-enriched pathways were the following: eukaryotic translation elongation, Translation initiation, Translation termination, SRP-dependent cotranslational targeting to membrane, and tRNA charging. De-regulated pathways included myelination signaling, extracellular matrix organization, and GP6 signaling pathways. In the BTBR background, pathway analysis similarly identified tRNA charging as the top enriched function (Figure 2B), again pointing to altered translation dynamics. Other upregulated pathways included response of EIF2AK1 (HRI) to heme deficiency and EIF2 signaling, which is tightly linked to global translation rates under metabolic challenge. Among all altered pathways, the downregulation of myelination signaling is noteworthy, as it suggests potential deficits in oligodendrocyte function or white matter development. When directly comparing pathway activation patterns between the two genetic backgrounds (Figure 2C), we observed a high degree of concordance, particularly in the upregulation of core translational pathways such as tRNA charging and EIF2 signaling. This suggests that the enu2 mutation triggers a shared molecular response involving potentiated translation capacity, likely reflecting compensatory mechanisms to counter metabolic dysregulation. Notably, additional overlapping pathways included the response of EIF2AK4 (GCN2) to amino acid deficiency, Selenoamino acid metabolism, and eukaryotic translation initiation, all consistently enriched in both backgrounds. These shared activations highlight a conserved transcriptional signature centered on metabolic stress and translational adaptation.

### 3.3. Increasing Attention Loading Also Highlights Deficits in C57^enu2^ Mice

**Figure 3:**
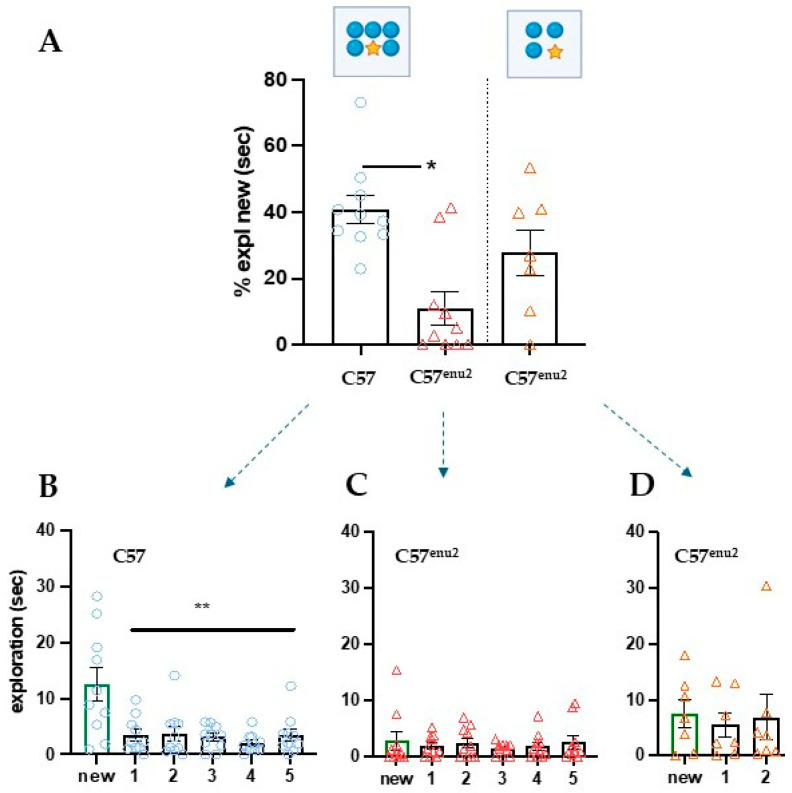
Memory deficits in C57^enu2^ mice in the Identical Object Task (IOT) under a high information load (4 and 6 objects in upper panel). (**A**) Bar graph showing the percentage of novel object exploration by C57^enu2^ mice in the 4-IOT and 6-IOT conditions. A high memory load (6-IOT) exacerbates information processing deficits in C57^enu2^ mice compared to control C57 mice (HF (3, 27) = 9.083, *p* = 0.0107). Bar charts report exploration of single objects in IOT. (**B**–**D**) Statistical analysis shows that only WT C57 mice explored the novel object significantly more than all familiar objects in the 6-IOT ((WT F (6, 10) = 19.25, *p* = 0.0017; n = 10); C57^enu2^ (F (6, 10) = 2.976, *p* = 0.7037; n = 10)), while C57^enu2^ mice failed starting from the 4-IOT (F (4, 7) = 1.087, *p* = 0.7802; n = 7). Values expresses as mean ± SEM. * = *p* < 0.05; ** = *p* < 0.01.

The presence of a common mRNA dysregulation pathway in the pFC suggests that differences in cognitive performance between the two mutated strains may be more quantitative than qualitative. To challenge the memory loading limits in C57^enu2^ mice, we used the IOT with an increase in the number of identical objects (Figure 3). Based on animals’ natural curiosity to explore new objects, IOT tests processing load by increasing the number of objects to pay attention to (four and six objects in our experiment). While regular locomotor activity and general exploration occurred during the test session (Figure S1), a significant difference between C57 and C57^enu2^ was observed in the recognition index (Figure 3A) only when six objects were tested (H (3, 27) = 9.083, *p* = 0.0107). Interestingly, when evaluating the time spent exploring the new vs. the old objects, only WT mice succeeded in the task (F (6, 10) = 19.25, *p* = 0.0017; n = 10), while C57^enu2^ did not discriminate between objects in the four- or six-object task, as shown in Figure 3C,D (four objects F (4, 7) = 1.087, *p* = 0.7802; n = 7); six objects (F (6, 10) = 2.976, *p* = 0.7037; n = 10). These results suggest that four objects might be a borderline number for C57^enu2^ attention load; this is also indicated by the individual variability of performance in C57^enu2^ mice subjected to 4-IOT (Figure 3C,D). Given the difficulty C57^enu2^ had in performing the recognition test with just four objects, and especially with six identical objects, the animal groups were not exposed to the Different Object Task (DOT) proposed as the second phase of the DOT/IOT test.

## 4. Discussion

The main finding of the present study was the alteration of aminoacyl-tRNA synthetases and eukaryotic translation factors, indicating transcriptional modulation of genes involved in protein translation in the two mouse models of severe HPA, regardless of their different genetic backgrounds. Indeed, of the 391 DEGs in C57 and of the 306 in BTBR, 62 were shared between strains and displayed a strong consistency in directionality. These commonly altered genes pointed toward a common molecular response involving metabolic stress and impaired protein synthesis. These transcriptional alteration patterns may be caused by a disrupted amino acid balance due to increased blood and brain Phe.

Indeed, evidence from both clinical and animal studies suggests that cerebral protein synthesis is inversely correlated with plasma Phe levels [17], and PKU mice of the two backgrounds are characterized by similarly severe plasma and cerebral HPA levels [18]. This phenotype appears to be in contrast with the direction of pathway changes identified in our study, possibly because the increased transcription of protein synthesis-related factors may represent a transcriptional response associated with altered protein synthesis under conditions of chronic hyperphenylalaninemia.

We also identified a downregulation of gene programs related to myelination in enu2 mutants regardless of the genetic background (Figure 2). Abnormal myelination has been repeatedly demonstrated in PKU patients. Moreover, in a histological assessment of the brain from untreated, intellectually disabled adult PKU patients, reduced staining was especially evident in areas that undergo significant postnatal myelination, such as the axonal connections to the pFC [[19] for review]. Interestingly, previous findings in BTBR^enu2^ mice indicated altered expression dynamics of myelin basic protein (MBP) mediated by changes in both transcriptional- and microRNA-related post-transcriptional regulation [20].

Shared neurobiological phenotypes are expected in genetic models derived from the same point mutation, supporting the value of these models for preclinical research [21]. On the other hand, published reports have documented differences in neural and behavioral phenotypes associated with the enu2 mutation in the two genetic backgrounds [12].

In this context, it is noteworthy that only three of the commonly modulated genes—Nup62, Psph, and Arhgef2—showed opposite regulation patterns between the two genetic backgrounds, suggesting a potential contribution to strain-specific phenotypic differences. Nup62 encodes a core component of the nuclear pore complex and plays a key role in nucleocytoplasmic transport, including the export of mRNAs required for protein translation, a process that may be particularly relevant under conditions of altered protein synthesis such as chronic HPA [22]. Psph, which encodes phosphoserine phosphatase, is involved in the final step of L-serine biosynthesis, a pathway critical for normal brain development and function. Mutations affecting this enzyme have been described in human metabolic disorders associated with severe neurological symptoms, such as Williams Syndrome, highlighting the importance of serine metabolism in neurodevelopment and behavior [23]. Finally, Arhgef2 encodes a Rho guanine nucleotide exchange factor involved in G-protein-coupled signaling and cytoskeletal dynamics and can lead to midbrain and hindbrain abnormalities. Variants of Arhgef2 have been associated with abnormal neuronal morphogenesis and impaired brain development, including alterations affecting midbrain and hindbrain structures [24]. Differential regulation of this gene may thus influence neural connectivity and functional vulnerability in a background-dependent manner.

Regarding the specific behavioral phenotype observed in the two different backgrounds, it was reported that while adult BTBR^enu2^ mice do not discriminate between an object explored 24 h before and a novel object, adult C57^enu2^ mice do discriminate between the two [25]. These data reflect a severe memory and learning deficit in BTBR^enu2^ but not in PKU mice with a C57 genetic background. Accordingly, C57^enu2^ mice have shown deficits only in a specific and demanding task, such as the Morris Water Maze [26], confirming a less compromised cognitive phenotype. It is worth pointing out that the Morris Water Maze is a hippocampus-dependent test not requiring a functioning pFC. In contrast, the results of our genome-wide transcriptome analysis suggest a deficit in pFC-dependent functioning shared between strains. Since evidence of cognitive pFC-dependent impairments was demonstrated only in BTBR^enu2^ mice [5,6,7,8], BTBR^enu2^ mice were not further tested in the object recognition test (according to the principles of the 3Rs, in particular Reduction, which requires minimizing the number of animals used), and we evaluated working memory using the IOT test with a loading of processing information only in adult C57^enu2^ mice. This procedure, designed to explore object memory span in mice [13,16], enables the evaluation of pFC-dependent performance under increasing attentional load. Six objects represent a processing overload in the IOT test for C57^enu2^ mice (Figure 3). These findings also highlight, for the first time, a pFC-dependent deficit in C57^enu2^ mice, possibly related to the mRNA dysregulation pathway fostered by HPA also existing in the pFC of this strain.

It is important to recognize that the pFC is one of the brain regions most susceptible to HPA-related deficits. In fact, Diamond et al. [1] demonstrated that children with PKU, even when treated early, show specific impairments in working memory and inhibitory control—functions primarily managed by the pFC. Leuzzi et al. [2] also confirmed executive dysfunctions in PKU patients with normal IQ. Moreover, Christ et al. [3] observed abnormal activation and reduced functional connectivity in the pFC during working memory tasks in early-treated PKU individuals using fMRI. Furthermore, longitudinal data indicate that variability in early-treated PKU IQ values cannot be entirely explained by either developmental or adult quality of metabolic control [27,28]. Also, more recent neuroimaging and longitudinal studies document pFC and cognitive dysfunctions in ETPKU patients, often linked to lifelong metabolic control [29,30].

The current findings indicate that the phenotypic difference in how HPA affects the two strains is quantitative rather than qualitative. The C57 background offers greater resilience and adaptive capacity in response to the same biochemical changes, albeit through unknown mechanisms [12]. On translational grounds, together, BTBR and C57 enu2 models offer a spectrum of phenotypes that may better reflect the variability in the severity of HPA-induced neurological deficits reported in clinical studies. They may help in exploring more subtle alterations associated with the disease and identifying potential further markers for treatment assessment in both mature and developing brains. The transcriptomics analysis would benefit from validation through the examination of candidate proteins, to verify the final effects of the dynamic modifications.

## 5. Conclusions

The present study demonstrates that developmental HPA induces a highly conserved pattern of transcriptomic alterations in the pFC across distinct genetic backgrounds. Specifically, the enu2 mutation consistently affected genes involved in amino acid metabolism and protein synthesis, suggesting a shared adaptive response to metabolic stress. Despite this substantial overlap, some pathways appear to exert predominant functional effects depending on the genetic background. Myelination-related pathways were more prominently downregulated in BTBR^enu2^ mice, whereas in C57 ^enu2^ mice, alterations mainly involved transcripts associated with eukaryotic translation initiation and elongation, indicating a global effect on protein synthesis.

Functionally, behavioral testing confirmed that C57^enu2^ mice exhibit pFC-dependent working memory deficits under a high information load, extending to this strain a cognitive phenotype previously reported only in BTBR^enu2^ mice. This convergence of molecular and behavioral data supports the hypothesis that HPA triggers core pFC dysfunctions regardless of strain, though their magnitude and expression are shaped by background-dependent adaptive mechanisms.

Altogether, these findings highlight the relevance of comparative studies between enu2 models for dissecting the mechanisms underlying differential vulnerability and resilience to HPA. We consider the strain-dependent susceptibility to HPA status in the two different PKU murine models to be consistent with the clinical variability in HPA observed in ETPKU patients, particularly in terms of variability in adult cognitive outcomes beyond average Phe levels. 

A main strength of this study is the comparative transcriptomic analysis across two genetic backgrounds, which enabled the identification of conserved pFC molecular signatures associated with severe HPA. Limitations include the relatively small sample size, the lack of RT-qPCR/protein level validation, the fact that behavioral testing was not replicated in the BTBR^enu2^ strain (impeding a direct strain-by-strain behavioral comparison), and the absence of direct clinical correlations. We are confident that the present preclinical approach may contribute to the identification of potentially patient-specific targets for interventions aimed at preserving prefrontal functioning in PKU.

## Data Availability

The original contributions presented in this study are included in the article/Supplementary Material. Further inquiries can be directed to the corresponding author.

## Supplementary Materials

The following supporting information can be downloaded at: https://www.mdpi.com/article/10.3390/cells15030227/s1, Table S1: Differentially expressed genes (DEGs) in the pFC of mutant mice versus WT controls (C57 background); Table S2: Differentially expressed genes (DEGs) in the pFC of PKU mice versus WT controls (BTBR background); Figure S1: No differences between C57 and C57^enu2^ in OF activity.

## Abbreviations

The following abbreviations are used in this manuscript:

PKU	phenylketonuria
HPA	hyperphenylalaninemia
pFC	prefrontal cortex
Phe	phenylalanine
Tyr	tyrosine
ETPKU	early-treated PKU
IQ	intellectual quotient
BTBR	Black and Tan Brachyury
C57	C57Bl/6JRj
IOT	identical object task
WMC	working memory capacity
DEGs	differentially expressed genes
MBP	myelin basic protein
