# Supplementary material for: Phenylketonuria Alters the Prefrontal Cortex Genome-Wide Expression Profile Regardless of the Mouse Genetic Background
Source: Cells. 2026 Jan 24;15(3):227. doi: 10.3390/cells15030227 (PMC12896771; doi:10.3390/cells15030227)

Table S1. Differentially expressed genes (DEGs) in the pFC of ENU2 mice versus WT controls (C57 background). The table lists genes differentially expressed in ENU2 compared to WT mice (adjusted p-value < 0.05) as identified by mRNA-Seq. For each gene, the gene symbol, adjusted p-value (Padj), log2 fold change, and direction of regulation in ENU2 mice are reported. Positive Log2FoldChange values indicate upregulation in ENU2, whereas negative values indicate downregulation. A total of 65 genes were differentially expressed, of which 41 were upregulated and 24 downregulated in ENU2 compared to WT mice. Data were obtained from mRNA-Seq analysis of prefrontal cortex samples from 2-month-old male and female mice.

DEGs in C57: ENU2 vs WT

Gene Symbol	Padj Value	Log2 Fold Change	Direction in ENU2
Slc7a3	1,40E-16	1,53	UP
Atf5	4,79E-11	0,77	UP
Slc7a5	5,62E-07	0,42	UP
Nup62	5,62E-07	0,43	UP
Sars1	3,23E-05	0,31	UP
Itga7	4,88E-05	-0,62	DOWN
Asns	1,25E-04	0,42	UP
Cars1	1,42E-04	0,31	UP
Sesn2	1,42E-04	0,45	UP
Parvg	1,42E-04	0,73	UP
Gnptab	3,24E-04	-0,51	DOWN
Aars1	3,28E-04	0,31	UP
Yars1	3,33E-04	0,27	UP
Npas4	3,72E-04	1,23	UP
Rpl5	4,49E-04	0,24	UP
Slc3a2	6,63E-04	0,24	UP
Cox7a2l	1,37E-03	0,23	UP
Eef1a1	2,54E-03	0,23	UP
Samd4	2,66E-03	-0,27	DOWN
Cartpt	2,66E-03	-1,21	DOWN
Slc1a4	3,37E-03	0,30	UP
Cdr2	4,55E-03	-0,47	DOWN
Dmp1	5,44E-03	-0,46	DOWN
Slc12a4	5,44E-03	-0,43	DOWN
Mthfd2	1,00E-02	0,86	UP
Rpl10	1,22E-02	0,29	UP
Serpina3n	1,22E-02	-0,58	DOWN
Kcng4	1,32E-02	-1,03	DOWN
Gas2l3	1,32E-02	-1,11	DOWN
Adcyap1	1,32E-02	-0,45	DOWN
Nars1	1,32E-02	0,30	UP
Metap2	1,32E-02	-0,19	DOWN
Gars1	1,32E-02	0,25	UP
Rps24	1,34E-02	0,27	UP
Shmt2	1,36E-02	0,39	UP
Plekhg3	1,36E-02	-0,43	DOWN
Rpl4	1,36E-02	0,19	UP
Herpud1	1,36E-02	0,27	UP
Apaf1	1,54E-02	0,44	UP
Mars1	1,57E-02	0,26	UP
Bcat1	1,59E-02	0,31	UP
Tfrc	1,81E-02	-0,39	DOWN
Slc16a3	1,97E-02	-0,76	DOWN
Rpl21	2,05E-02	0,29	UP
Car2	2,35E-02	-0,27	DOWN
Trim66	2,44E-02	0,36	UP
Lars1	2,53E-02	0,24	UP
Eef1g	2,75E-02	0,22	UP
Plxdc1	2,75E-02	0,42	UP
Ptgs1	2,90E-02	-0,32	DOWN
Col6a2	2,90E-02	0,51	UP
Pip5kl1	2,93E-02	-0,45	DOWN
H2-Eb1	3,43E-02	-1,36	DOWN
Ntng1	3,76E-02	-0,65	DOWN
Tmod1	3,77E-02	-0,24	DOWN
Slc7a1	4,09E-02	0,35	UP
Peg10	4,50E-02	-0,38	DOWN
Hadhb	4,67E-02	-0,21	DOWN
Rps7	4,86E-02	0,25	UP
Dner	4,98E-02	-0,23	DOWN
Dusp1	4,98E-02	0,70	UP
Aldh1l2	4,98E-02	0,47	UP
Mroh5	4,98E-02	0,66	UP
Cyr61	4,98E-02	1,23	UP
Cry2	4,98E-02	0,14	UP

Table S2. Differentially expressed genes (DEGs) in the pFC of ENU2 mice versus WT controls (BTBR background). This table reports genes significantly differentially expressed in ENU2 mice compared to WT mice on the BTBR genetic background (adjusted p-value < 0.05). Columns indicate gene symbol, adjusted p-value (Padj), log2 fold change (Log2FC), and direction of regulation in ENU2 mice. Positive Log2FC values denote genes upregulated in ENU2 mice, while negative values represent downregulated genes. A total of 42 genes were differentially expressed, of which 27 were upregulated and 15 downregulated in ENU2 mice. Data were obtained from mRNA-Seq analysis of prefrontal cortex samples from 2-month-old male and female.

DEGs in BTBR: ENU2 vs WT

Gene Symbol	Padj Value	Log2FoldChange	Direction in ENU2
Cars1	2,00E-11	0,81	UP
Slc7a3	4,13E-09	2,04	UP
Yars1	1,02E-07	0,41	UP
Aars1	8,37E-07	0,39	UP
Chac1	6,77E-06	1,32	UP
Mthfd2	1,38E-05	1,29	UP
Rabggtb	1,22E-04	0,40	UP
Sesn2	1,67E-04	0,73	UP
Slc7a5	1,67E-04	0,59	UP
Sars1	2,61E-04	0,33	UP
Klhl33	7,43E-04	0,93	UP
Iars1	7,43E-04	0,41	UP
Nup62	1,10E-03	0,53	UP
Xpot	1,65E-03	0,27	UP
H2-Ab1	1,98E-03	-4,23	DOWN
Slc1a4	2,98E-03	0,47	UP
Psph	6,66E-03	0,81	UP
Lars1	9,53E-03	0,32	UP
Sdc4	9,59E-03	-0,61	DOWN
Mars1	1,23E-02	0,36	UP
Atf4	1,23E-02	0,51	UP
Eif4b	1,42E-02	0,23	UP
Wnt6	1,42E-02	-3,00	DOWN
Eif4ebp1	1,49E-02	1,17	UP
Pmp22	1,83E-02	-0,58	DOWN
Slc12a4	1,84E-02	-0,69	DOWN
Lamc2	1,98E-02	-1,07	DOWN
Noc2l	2,04E-02	0,22	UP
Asns	3,21E-02	0,58	UP
Serbp1	3,60E-02	0,23	UP
Lrrc25	3,75E-02	-2,51	DOWN
Prr5	3,91E-02	-0,75	DOWN
Itga7	3,91E-02	-0,82	DOWN
Sult1a1	3,92E-02	-0,85	DOWN
Gars1	3,92E-02	0,41	UP
Eif3c	4,00E-02	0,21	UP
Scrg1	4,00E-02	-0,59	DOWN
Zbtb7b	4,25E-02	-0,41	DOWN
Nars1	4,49E-02	0,44	UP
Adamts1	4,75E-02	-0,58	DOWN
H2-Aa	4,75E-02	-4,08	DOWN
Serpind1	4,75E-02	-1,92	DOWN

Figure S1. No differences between C57 and C57enu2 were find in OF parameters: (A)Distance moved; (B) Velocity; (C) Time spent in immobility; (D) Total exploration; (E) Time spent in the center of the apparatus. Values are expressed as mean± SEM.

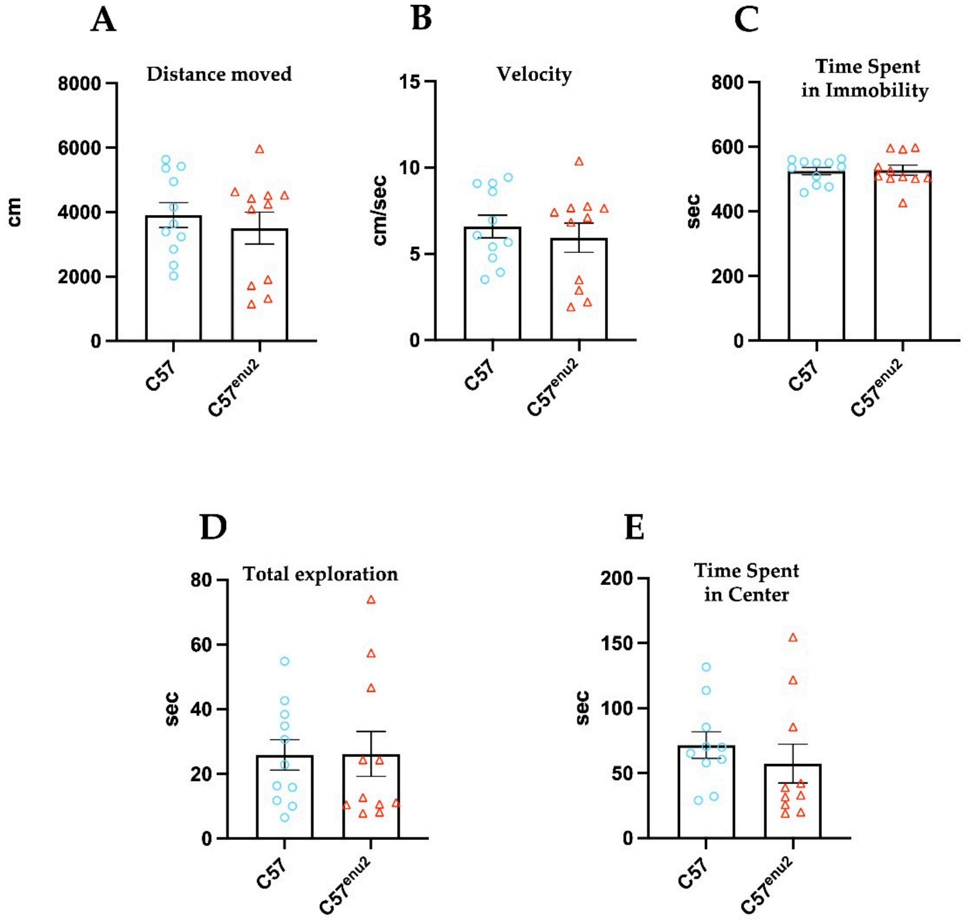

Supplement: Supplementary file 1 [file cells-15-00227-s001.zip › cells-4028236-supplementary.pdf]
